# Machinery Safety and Ergonomics: A Case Study Research to Augment Agricultural Tracklaying Tractors’ Safety and Usability

**DOI:** 10.3390/ijerph18168643

**Published:** 2021-08-16

**Authors:** Davide Gattamelata, Leonardo Vita, Mario Fargnoli

**Affiliations:** 1 Italian Workers’ Compensation Authority (INAIL), Via Fontana Candida 1, Monte Porzio Catone, 00078 Rome, Italy; d.gattamelata@inail.it (D.G.); l.vita@inail.it (L.V.); 2 DIMA, Faculty of Civil and Industrial Engineering, Sapienza University of Rome, Via Eudossiana 18, 00184 Rome, Italy

**Keywords:** machinery safety, ergonomics, human behavior, agricultural tractors, roll-over protective structure (ROPS), partial assistance system (PAS), reverse engineering

## Abstract

Occupational Health and Safety (OHS) in agricultural activities is an issue of major concern worldwide notwithstanding the ever stricter regulations issued in this sector. In particular, most accidents are related to the use of tractors and the main causes of this phenomenon are due to the lack of rollover protective structures (ROPSs). This happens especially when tractors are used in particular in-field operations that are characterized by limited clearances between tractor and crop rows so that farmers usually use tractors without ROPS (e.g., dismounting it). To solve such a problem, foldable protective structures (FROPSs) have been proposed, which should augment the operator’s protection. However, FROPS’s conventional solutions underestimate the operators’ risk-taking behavior and the widespread misuse of FROPS due to the efforts needed to operate it. The current study aims at contributing to the improvement of the latter issue proposing the development of a novel approach for the implementation of partial assistance systems (PASs) that can reduce the physical effort of the operator when raising/lowering the FROPS. The proposed methodology, which is based on a reverse engineering approach, was verified by means of a practical case study on a tracklaying tractor. Results achieved can contribute to expanding knowledge on technical solutions aimed at improving the human-machinery interaction in the agricultural sector.

## 1. Introduction

**Figure 1:**
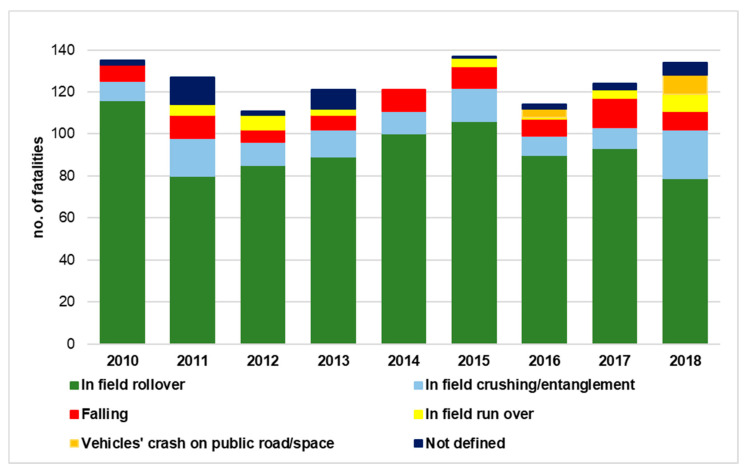
Occupational fatal accidents that occurred in Italy in agriculture (data elaborated from [5]).

Occupational safety is a relevant aspect of the social pillar in sustainable development: hence, safety research aimed at reducing occupational accidents can be valuable for the practical implementation of sustainability at the company level. In such a context, it has to be remarked that a safety problem of major concern worldwide is related to the use of agricultural tractors. Actually, the unsafe use of this work equipment as well as the use of unsafe tractors, i.e., tractors that are not in compliance with safety protection requirements, are the cause of a large number of serious and fatal accidents in most countries [1,2,3]. This is particularly true with respect to the roll-over risk [4]: for example, considering the Italian context, information collected by the national observatory of the Italian Workers Compensation Authority (INAIL)—Research Department [5] on fatal accidents related to the use of tractors confirms that most fatalities are due to roll-over. As shown in Figure 1, the average value of fatalities due to in-field roll-over in the period 2010–2018 is 74.7%.

Additionally, it has to be considered that most fatalities occurred when the roll-over protective structure (ROPS) was not installed, or when it was disabled, as in the case of foldable ROPSs (FROPSs): the incidence of these cases is very large as reported by different authors according to which from 30% to 50% of fatalities occurred due to the misuse of tractor FROPS [6,7,8].

To reduce such a phenomenon, a considerable effort has been made by public authorities over the years, both promoting the installation of ROPSs on old tractors (e.g., by means of retrofit campaigns [9,10] or the provision of technical guidelines to support retrofitting [11]), as well as introducing mandatory requirements for the ROPSs’ manufacturing and testing. Indeed, in many countries these obligations have significantly reduced the fatal accidents resulting from roll-over, demonstrating the effectiveness of this safety device combined with the use of seatbelts [12,13].

However, despite such improvements, a difficulty remains when considering tractors that are usually used in works where the clearances (both in height, and in width) between tractor and crop rows are smaller than the usual ones, and the protective structure represents a hindrance for some works requiring the use of FROPSs. In these cases, the use of tractors with unfolded ROPS is allowed during particular in-field operations only (e.g., in vineyards, glasshouses, or orchards), since the low speed of these activities can reduce the risk of roll-over anyway. Moreover, they can be folded down also for tractor storage. On the contrary, they must always be kept upright when the tractor is used for other activities (i.e., works where the ROPS’ dimensions do not represent a physical hindrance) and during transfers (e.g., from one operation site to another, or from the storage place to fields). Indeed, the most common behavior of farmers using this type of machinery consists of leaving the ROPS always unfolded since the folding/unfolding operations require certain stress and cause an interruption of working activities [14,15]. Such unsafe behavior is the cause of numerous serious and fatal accidents worldwide, since, in the case of roll-over, if the ROPS is unfolded the operator is fully unprotected. 

Hence, on the one hand, work activities in contexts where the space is limited by different types of obstacles require the use of tractors equipped with FROPSs, which is very diffused also among non-professional farmers [16]. It has to be noted that these tractors are not only those characterized by a reduced track width (i.e., the so-called narrow-track tractors) but also other models equipped with both tires and tracklaying solutions (i.e., when the tractor is propelled and steered by endless tracks [17]). On the other hand, albeit safety regulations from both the manufactures’ and entrepreneurs’ points of view stress the necessity to take into account the “reasonably foreseeable misuse” or readily predictable human behavior of machinery users [18], conventional solutions for foldable ROPS underestimate the operators’ risk-taking behavior, as well as the widespread FROPS misuse due to the efforts needed to operate it [14,19]. 

In the literature, several studies have dealt with this problem proposing technical solutions aimed at preventing the incorrect behavior of operators. For example, Silleli et al. [6,20] developed an automatically deployable anchor mechanism to prevent continuous rolling during sideways roll-over, protecting the operator from getting injured in case of an overturn. Such a system, which in brief consists of a deployable bar mounted on the top of the ROPS, can provide an increased clearance zone at the lateral direction in case of overturning, allowing the reduction of the ROPS’ height that makes orchard and vineyard work easier.

Ballesteros et al. [21] designed an automatically deployable front-mounted ROPS, which is capable of being deployed in height and width, as well as being locked in its operative position thanks to airbag inflators activated by roll-over sensors. Such a solution, which was named Ejectable in Two Dimensions ROPS (E2D-ROPS) and does not require the intervention of the tractor operator, was further developed [22], while other studies have proposed similar automated deployable ROPS solutions [23,24,25,26]. These solutions can certainly augment the safety level of the operators since they prevent them from making a decision on whether and when to fold/unfold the ROPS. However, it has to be noted that these solutions are mainly destined to equip new tractors, as they are unlikely to be adapted to already-in-use tractors because of the costs and technical efforts required for the installation. 

Other research addressed the problem from an ergonomics standpoint. In particular, Cremasco et al. [14] highlighted that FROPS solutions hardly take into account the users’ perspective since the human–tractor interaction in terms of FROPS’ reachability and comfort in use is neglected. Actually, investigating the behavior of 20 farmers in dealing with 16 models of tractors equipped with two-posts rear FROPS, they noted that unsafe, uncomfortable, and awkward behaviors were mainly due to the FROPS technical features, suggesting that further research on the development of more human-centered solutions is necessary. Differently, Pessina et al. [27] investigated the effort needed to unfold/fold FROPSs examining 19 different tractors equipped with two-posts front FROPSs. The authors pointed out the need to reduce the handling load the tractor operators have to deal with when using FROPSs. 

In addition, it is worth mentioning other studies providing solutions that avoid the intervention of the operator, such as the compact roll-over protective structure (CROPS), i.e., a modified four-posts ROPS for narrow-track tractors developed by Italian researchers from the Italian Workers Compensation Authority (INAIL) in collaboration with the academia, which provides a protective shell on the operator while reducing the height and width of the system [28,29]. Despite their effectiveness in eliminating the risk of FROPS misuse, these solutions can be applied to a limited number of tractor models due to their technical features. Moreover, it should also be considered that if the ROPS’ type of a tractor already on the market is changed, the tractor’s homologation needs to be obtained again as in most countries (e.g., in the European Union), tractors are considered vehicles and thus undergo specific regulations and approval procedures to be used in public areas such as roads [30]. Hence, their technical and economic feasibility in retrofitting/upgrading already-in-use models is very limited. 

Moreover, from interviews with ROPS’ manufacturers on the type of aftermarket ROPS purchased by farmers to update their tractors, it emerged that the purchase of automated systems for the FROPS handling is very rare due to the high cost of this type of solution.

Finally, it has to be outlined that from the technical normative point of view, OECD Codes provide technical information on the FROPS’ actuation forces only for narrow-track wheeled tractors or tractors with tracks instead of wheels, while such ergonomic issues for other types of tractor-FROPS combinations (i.e., tractors mounted with endless tracks) are not foreseen. Thus, ROPS manufacturers have no reference frameworks when developing supportive systems for front-mounted FROPSs destined to tracklaying tractors (both new and aftermarket vehicles), since the provision of a practical procedure to evaluate FROPS’ actuation forces, as well as the feasibility of partial assistance systems and their interaction with the operator is missing in these cases.

Based on these considerations, despite representing an important issue in the field of agricultural tractor safety [15,18], it is evident that the problem of retrofitting/upgrading tractors with FROPS solutions is scarcely addressed, and also ergonomic issues related to the operator-FROPS interaction are not investigated sufficiently. The current study aims at reducing such research gaps proposing the development of a novel approach for the implementation of partial assistance systems (PASs) that can reduce the physical effort of the operator when raising/lowering front-mounted foldable ROPS, diminishing in this way its misuse. In more detail, the research work carried out followed a reverse engineering approach, which started from the analysis of ergonomic issues related to the movements that operators have to make when folding/unfolding front-mounted FROPSs. To reduce this stress facilitating the protective structure handling, the interaction FROPS-operator was analyzed to determine the loads needed for the lowering/raising operations practically. Accordingly, a procedure for the proper dimensioning and selection of a partial assistance device capable of reducing the efforts of the operator was developed and its feasibility was verified by means of physical prototyping and practical testing on a tracklaying tractor. 

The remainder of the article is as follows: in the next section, the background analysis is provided focusing on the safety requirements of foldable protective structures and their grasping points. Then, in Section 3, the research approach is summarized, while its practical application to the development of a partial assistance system destined to equip the protective structure of a tracklaying tractor is presented in Section 4. The discussion of results is reported in Section 5, while Section 6 concludes the paper addressing further research goals.

## 2. Background Analysis

In the European Union for tractors put on the market from 1974, the European directives 74/150/EEC and 2003/37/EC were used. These directives were repealed by the Regulation (UE) 167/2013 [30], which updated the legislative framework for tractor safety and confirmed as a reference for ROPS’ testing criteria provided by the Organization for Economic Co-operation and Development (OECD) standard codes [17]. Briefly, these codes indicate that roll-over protection structures are safety devices aimed at ensuring an unobstructed space inside them (i.e., a safety zone called “clearance zone” or “deflection limiting volume” (DLV), depending on the tractor type and related standard code) large enough to protect the operator in case of tractor’s overturning or tip-over. Such requirement is valid both for ROPSs installed by the tractor manufacturer (i.e., before the tractor is put on the market) and for those devices put on the market separately and intended for such vehicles. To verify compliance with this requirement, the use of the OECD standard Codes as technical reference documents is foreseen. In particular, according to the OECD Code no. 6 [17], narrow-track tractors are characterized by: a reduced height from the ground (ground clearance of not more than 600 mm beneath the lowest points of the front and rear axles); a reduced track width (fixed or adjustable minimum track width with one of the axles less than 1150 mm fitted with tires or tracks of a larger size); a mass (i.e., the unladen mass of the tractor, including the roll-over protective structure and tires or tracks of the largest size recommended by the manufacturer) spanning from 400 kg to 3500 kg. To be precise, OECD Code no.6 is related to criteria for the testing of front-mounted roll-over protective structures on narrow-track agricultural and forestry tractors, while rear-mounted ROPSs for these types of tractors are treated in OECD Code no. 7 [17]. Besides these technical parameters, which are destined to tractors’ and ROPS’ manufacturers, ergonomic issues aimed at taking into account the users of ROPSs’ point of view are addressed partially. In fact, the recent updates of the OECD Code no. 6 and no. 7 have introduced an additional procedure for testing FROPS solutions, which includes criteria for measuring loads in the manual operations of raising and lowering the FROPSs. In more detail, Section 3.8 of the OECD Code no. 6 (and Section 3.7 of the OECD Code no.7) introduces additional requirements for FROPS, where the “grasping area” is intended as the portion of the FROPS where the operator is allowed to carry out the raising/lowering operations. This part is defined by the manufacturer and can include an additional handle fitted to the FROPS. Based on this, it is also worth mentioning the definition of both the “accessible part of the grasping area” (i.e., the area where the FROPS is handled by the operator during the raising/lowering operations), and the “accessible zone” (i.e., the volume where a standing operator can apply a force in order to raise/lower the FROPS).

**Figure 2:**
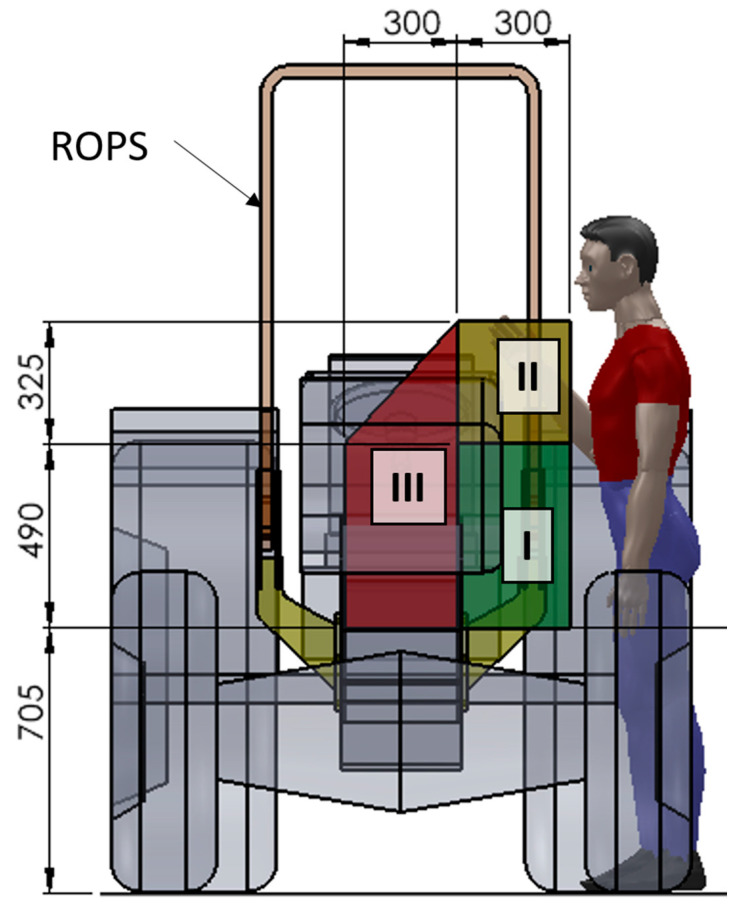
Scheme of the accessible zones of the grasping area considering a wheeled tractor where measures are expressed in mm (elaborated from [17]).

To carry out folding/unfolding operations safely, three different accessible zones are introduced, each characterized by a different amount of allowed force for raising/lowering operations (Figure 2):

Zone I: comfort zone;
Zone II: accessible zone without forward leaning of the body;
Zone III: accessible zone with forward leaning of the body.

These zones are defined with respect to the horizontal plane of the ground and the vertical planes tangent to the outer parts of the tractor, which limit the position or the displacement of the operator. According to the OECD Codes, the accessible area shall be considered as “the envelope of the different accessible zones”. The position and the movement of the operator are limited by those parts of the tractor representing an obstacle (i.e., the wheels represent an obstacle in sideward movements, while the operator can always move backwards) and defined by vertical planes tangent to the external edges of the obstacle. In Table 1 the limits related to the raising/lowering forces provided by the Code are reported.

It has to be noted that these limits represent the acceptable force for the actuation of the FROPS in relation to the different accessible zones: such values can be augmented up to 50% for lowering operations, while an increase up to 25% is allowed when the roll-bar is fully raised or fully lowered. However, these values do not take into account the real loads the operators have to deal with when raising/lowering the FROPS, as observed by Pessina et al. [27], who carried out practical tests of 19 different tractors equipped with two-pillars front FROPS. They argued that to be in compliance with these limits it is necessary to apply at least a partial assistance device to reduce the operator’s required force. This problem was outlined also by Franceschetti and Rondelli [31], who analyzed six two-post FROPS mounted in front of the driver on six narrow-track tractors with different mass and geometry. They brought to light that a proper definition of the force (torque) limits is related to both the ease of access to the FROPS grasping area and to the tractor geometry, while OECD Codes do not take into account that increasing the mass and dimensions of the tractor, the operator’s required efforts for lowering/raising the FROPS increase not only because of the heavier FROPS but also for the uncomfortable zone for these operations. Similarly, Vigoroso et al. [32], analyzing the main criticalities in handling rear-mounted FROPS by means of the involvement of a group of users, highlighted the necessity of providing human-centered solutions capable of reducing the effort and stress of operators to avoid FROPS misuse while working. Moreover, it is worth underlining that the above force limits and the related criteria for the FROPS lowering/raising grasping areas are addressed to two types of tractor-ROPS’ configurations only: the front FROPS on wheeled narrow-track tractors (OECD Code no. 6) and rear-mounted FROPS on narrow-track wheeled tractors (OECD Code no. 7). Differently, other types of configurations such as the front-mounted FROPS on tracklaying tractors are not considered in spite of the large diffusion of these models for multiple operations [33,34].

## 3. Research Approach

Based on the above considerations, a reverse engineering approach [3,13] was applied to develop a technical solution for a partial assistance device capable of satisfying the constructive requirements (i.e., the OECD Codes) and the practical needs of operators at the same time. Indeed, reverse engineering allows for a bottom-up analysis capable of providing solutions closer to the safety needs of workers [35]. As illustrated by Afeez et al. [36], the reverse engineering approach is largely used in the development process of products with ergonomic properties by means of the development of mathematical models. This allows engineers to directly involve the users in the process by collecting their feedback [37]. Accordingly, a four-phase approach was developed, characterized by the following activities:

- Preliminary analysis: analysis of constructive requisites (OECD Codes) and ergonomic issues (force and torque values for raising/lowering the ROPS).
- Concrete experience: practical tests of lowering/raising the ROPS to determine the operator’s position due to the tractor’s features and the FROPS’ folding angles during these operations, focusing the attention on the individuation of the grasping points due to both the operator’s habits and the tractor’s geometrical features.
- Modelling: development of a partial assistance system (PAS) by means of CAD tools and verification of geometrical and dynamical features of the system.
- Validation: prototyping and experimental testing of the system.

In particular, the modeling phase of our research approach consists of two main steps: functional analysis and CAD modeling, where the former is based on the forces’ balance that should be achieved in order to reduce the operator’s effort and discomfort while avoiding additional hazardous situations. With this goal in mind, a procedure to verify the compatibility of the PAS was developed taking into account the following aspects related to the interactions occurring between the PAS, the FROPS, and the operator:

1. Functional analysis, which is aimed at defining the interaction PAS-FROPS-operator.
2. Geometrical compatibility, i.e., the analysis of the physical and geometrical features of the system and its adaptation on a front-mounted FROPS for tracklaying tractors;
3. Dynamical compatibility, i.e., the analysis of the forces and the related moments involved in the FROPS handling to ensure safe and comfortable operations.

**Figure 3:**
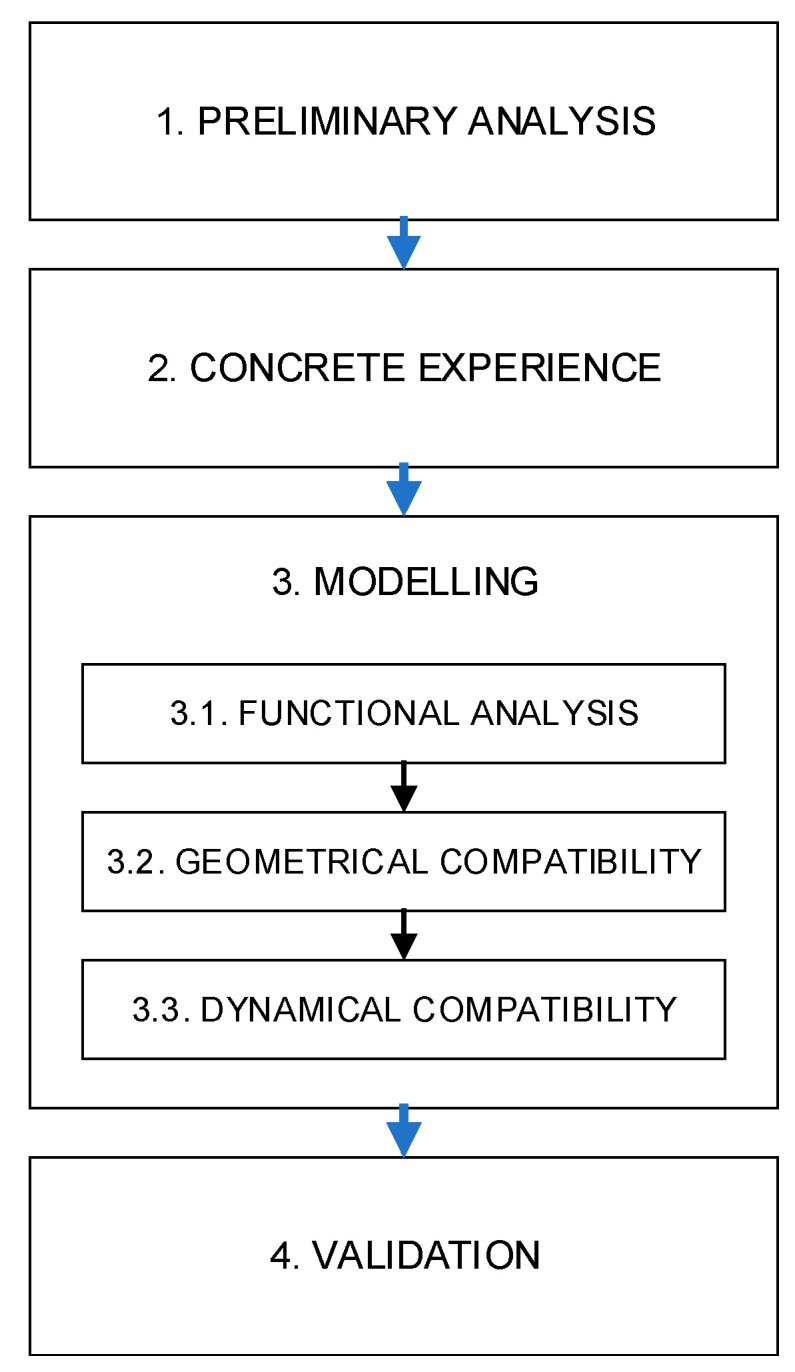
Scheme of the research approach.

In Figure 3, the research approach is summarized, while its implementation and verification are described by means of a practical case study.

## 4. Case Study 

Following the procedure shown in the previous section, a PAS for facilitating the raising/lowering operations of a two-posts front FROPS to be used on tractors propelled and steered by endless tracks was developed. The choice of using a tracklaying tractor was made because of the diffusion of this type of equipment not only in the agriculture and forestry activities, but also for other types of works such as earthmoving, and the numerous cases of accidents due to the lack of ROPS or to the FROPS misuse [12]. In particular, as a reference, a tracklaying tractor having the following characteristics was used: unballasted mass (i.e., the weight of the tractor excluding optional accessories but including coolant, oils, fuel, tools, plus the protective structure) 2.962 kg; overall length 2.78 m; wheelbase 1.53 m; track-width 1.66 m. 

### 4.1. Preliminary Analysis

The starting point of the analysis consisted in analyzing the technical features of a front-mounted FROPS for a tracklaying tractor. To understand the forces the operator needs to exert to handle the FROPS, its weight and length have to be determined.

**Figure 4:**
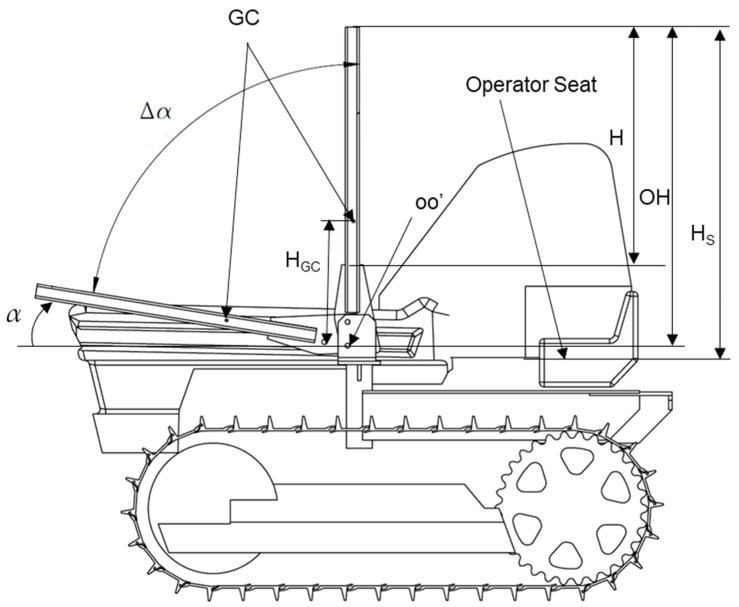
Main features of the FROPS: CG represents the center of gravity; H represents the length of the FROPS from the joint plate; OH represents the length of the FROPS’s foldable part from the hinge point O; H_S_ is the FROPS’ whole height from the seat; α is the folding angle, while Δα is its complementary angle.

With reference to Figure 4, it has to be noted that the whole length of the FROPS (H_S_) includes the length of the joint plate and that of the roll-bar (H): in other words, H_S_ represents the vertical distance between the horizontal plane tangent to the seat and the upper part of the FROPS in the safe configuration. Needless to say, the weight of FROPS depends on its dimensions, which are correlated to the tractor’s mass as per the OECD Codes. Consequently, the load to be balanced by the operator when raising/lowering the protective structure can be calculated as the torque, i.e., the moment that should be applied to the FROPS in its center of gravity (CG).

**Figure 5:**
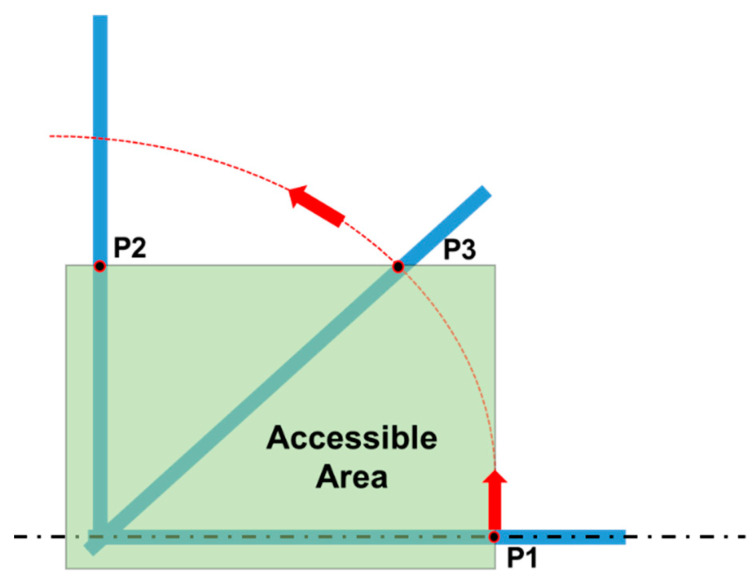
Grasping points in the accessible area shown from the side view (elaborated from [17]).

According to a market analysis and interviews with both ROPS’ manufacturers and users, it emerged that most diffused tracklaying tractors have an unballasted mass ranging from 2000 kg to 3500 kg, while the length of the FROPS’s foldable part (OH in Figure 5) varies from 1100 to 1400 mm. Therefore, to evaluate the loads the operators have to deal with, the center of gravity position (GC) has to be determined, which varies depending on the height of the FROPS’ roll-bar (i.e., the H_GC_ distance). More in detail, considering the above values the weight of the FROPS can be determined following the dimensioning rules proposed by the INAIL guidelines on ROPS’ retrofitting procedures [11] (Table 2). 

These data confirm that the FROPSs’ weight is considerable despite their handling is softened by the lever support represented by the hinge joint. Indeed, if considering the ISO 11228-1:2003 standard [38], which provides technical guidance on manual lifting tasks (i.e., moving an object from its initial position upwards without mechanical assistance), the reference mass (i.e., the mass considered appropriate for use with an identified user population) for non-repetitive operations is 25 kg with both hands and in the case of adult professional workers. Heavier objects can be lifted in special circumstances (up to 40 kg), which require specific training and information. Additionally, it has to be noted that when operating the FROPS the operator’s application force varies along with the roll-bar, based on the FROPS folding angle. Hence, the operator’s efforts needed to exert the FROPS can be considerable, augmenting not only the risk of accidents but also the exposure to musculoskeletal problems. According to the OECD Code no. 6, the manufacturer first shall evaluate the grasping area for FROPS’ raising/lowering and then verify that the applied load values do not exceed the limit values (see Table 1). These force limits should be considered valid in an optimal situation, i.e., when the operator handles the FROPS from a standing position in one of the grasping points suggested by the OECD Code no. 6. Moreover, as suggested by the ISO 11228-1:2003 standard, when lifting an object, it should be kept as close to the body as possible and both hands should be used, while stooped postures should be avoided. However, this situation hardly happens in practice due to the features of the tractor (i.e., the obstacle represented by the tracks) and the operators’ incorrect habits [14].

As far as the grasping area is concerned, the OECD Codes suggest that the force necessary to raise/lower the FROPS has to be determined considering different points that are within the accessible part of the grasping area. More in detail, with reference to Figure 5, these points should correspond to:

- the extremity of the accessible part of the grasping area when the FROPS is fully lowered (P1);
- the top of the accessible part of the grasping area when the FROPS is fully raised (P2);
- the position of P1 when reaching the top of the accessible part of the grasping area (P3).

Based on this, it is clear that the maximum forces in these points should not exceed the acceptable force limits reported in Table 1, while their practical definition can be made by measuring the torque needed to raise or lower the FROPS taking into account the distances of the grasping points from the hinge joint. 

### 4.2. Concrete Experience

**Figure 6:**
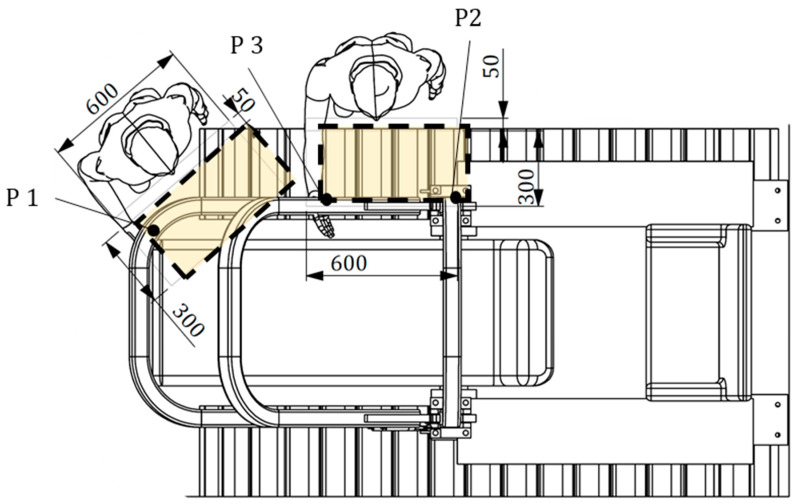
Top view of the tractor and positions of the operator when handling the FROPS (measures are expressed in mm).

The accessible area of a wheeled tractor (equipped with wheels or tracks, the front axle is not connected to the rear axle) differs from that of a tracklaying one, since in the latter case the operator does not stand in the space between the tires, but uses different grasping points along the roll-bar length (Figure 6), as emerged from practical tests that were carried out to determine the effective behavior of the operator when lowering/raising the FROPS. Such an analysis of the operator–FROPS interaction was performed in collaboration with a group of five different operators to better understand how they perform FROPS’ lowering/raising tasks in practice. It was found that during the lowering phase the operator changes the grasping point from the most uncomfortable position (i.e., close to the hinge joint) to the most comfortable one (i.e., the rest configuration). The opposite situation emerged when analyzing raising operations. It has to be noted that usually the rest configuration of FROPSs installed on tracklaying tractors corresponds to a folding angle of about 10° degrees. In fact, such a solution allows the use of FROPSs having a reduced length and weight considering the dimensions of the tractor’s bonnet, while ensuring a proper resistance of the protective structure. Starting from the rest position, the grasping point gets closer and closer to the hinge joint until the FROPS reaches the safe configuration. It was noted that during most operations the body of the operator is not close to the tractor’s chassis due to the obstacle represented by the tracks. Hence, unlike wheeled tractors, in our context the operator, when raising the FROPS from the rest configuration, starts from the outer part of the tractor to avoid the track since this position is felt as the most comfortable to raise the fully folded FROPS (Figure 6).

**Figure 7:**
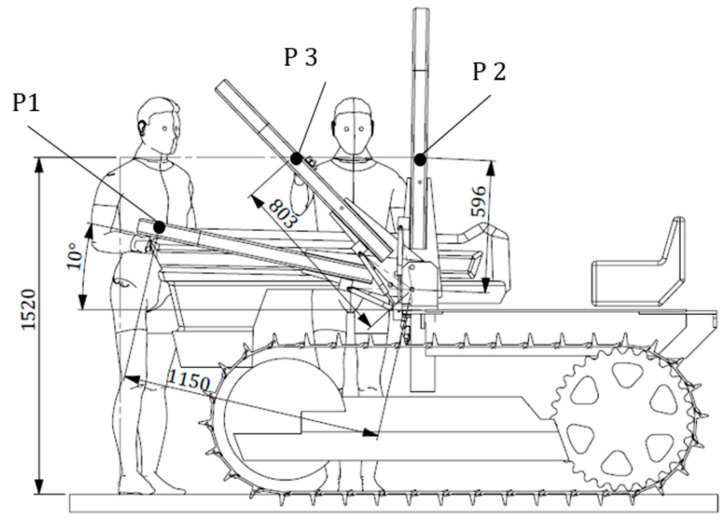
Positions of the grasping points to handle the FROPS (measures are expressed in mm).

Accordingly, in this context the accessible area differs from that of wheeled tractors and the grasping points mentioned above should be considered as follows: P1 corresponds to the position of the operator’s hand when starts the raising operation; P2 corresponds to the position of the operator’s hand when starts the lowering operation; P3 represents the position of the operator’s hand corresponding to a foldable angle of 45° degrees (Figure 7). 

In particular, taking into account the worst situations when handling the FROPS, the following grasping points were determined: P1 corresponds to the grasping point at the hedge of the roll-bar, which can be estimated between 90% and 95% of the roll-bar full length; P2 corresponds to the minimum height of the grasping point from the hinge joint of the FROPS just above the connection plate; and P3 corresponds to the position of the operator’s hand when the FROPS is half-raised (i.e., when the FROPS’ folding angle is equal to 45 degrees). It has to be noted that in most tracklaying tractors, as in our case study, the rest configuration of the FROPS corresponds to a folding angle α = 10°, because of the constructive features of the bonnet. In practice, the following measures of the grasping points were obtained:

- P1: a distance l_1_ = 1150 mm should be considered when raising the roll-bar from the rest configuration (FROPS lowered with a folding angle α = 10°).
- P2: a distance l_2_ = 596 mm should be considered when lowering the roll-bar from the safe configuration (FROPS fully raised).
- P3: a distance l_3_ = 803 mm should be considered when lowering the roll-bar, when the FROPS’ folding angle is α = 45°).

### 4.3. Modelling

The definition of a partial assistance system (PAS) to reduce the efforts of the operator when handling the FROPS was based on the consideration of a gas spring model, due to its widespread availability on the market and reduced costs. 

#### 4.3.1. Functional Analysis

In this step, the evaluation of the interactions between the PAS, the FROPS, and the operator was carried out taking into account the following elements:

- the PAS anchorage points;
- the support dimensions;
- the performances of the PAS.

The anchorage points’ determination is influenced by two main factors: the excursion of the gas spring and the presence of interferences of the lifting system with other tractor parts (e.g., bonnet, filters, etc.). This first analysis can be carried out practically, i.e., shifting the anchorage devices of the gas-spring around the roll-bar joints to verify whether the folded and raised positions of the roll bar are compatible with the stroke of the PAS elements. The interference with other parts of the tractor determines the need to change the FROPS configuration. In this case, also the anchor points and the PAS elements have to be changed. The selection of the PAS model also depends on the required forces to operate the FROPS roll-bar, avoiding the forces’ values being too high or insufficient, which can lead in both cases to additional risks for the operator. 

As far as the PAS performances are concerned, the forces involved in the raising and lowering tasks have to be investigated, depicting the following moments:

- M_W_, representing the moment of the weight force of the roll-bar, which varies depending on the horizontal distance of the gravity center (GC) from the axis of the hinge joint.
- M_PAS_, representing the moment of the PAS, which varies based on the PAS type and the forces it can exert on the roll-bar, considering the distance between the anchorage points and the hinge axis to determine the arm lever.

Accordingly, the efforts the operator has to deal with can be expressed as follows, distinguishing between the raising (M_RAI_ in Equation (1)) and the lowering (M_LOW_ in Equation (2)) tasks:

M_RAI_ = M_W_ − M_PAS_ (1)

M_LOW_ = M_PAS_ − M_W_ (2)

It should be considered that the weight force assumes the maximum value (M_WMAX_) in the rest configuration of the roll-bar and represents the force to be countered by the operator supported by the PAS to raise the FROPS. This is the reference value to select a proper PAS. On the one hand, a low force value makes the use of the PAS ineffective. On the other hand, the choice of a PAS capable of exerting an excessive force on the roll-bar can have a negative impact on the operator, mainly in the lowering phase (i.e., starting from the safe configuration when the FROPS is unfolded), as the operator can be hit by the FROPS. Hence, its proper dimensioning is crucial from the ergonomics and safety point of view.

The maximum force the operator can exert on the FROPS in a comfortable manner can be depicted by the values suggested by the OECD Code no. 6, indicating the limits of the acceptable force for the actuation of the FROPS (see Table 1). This assumption is made taking into account that the operator’s handling efforts are similar in the case of front-mounted FROPS, regardless of the tractor type. However, because of the different features of these types of tractors, the worst conditions should be considered. Hence, the accessible Zone II was always taken into account (see Figure 3) and consequently the acceptable force limit is F_OECD_ = 75 N (Table 1), adding the force limit augmentations foreseen by the OECD Code no. 6 in the following three situations:

(1) raising the roll-bar when the FROPS is fully lowered (rest configuration);
(2) lowering the roll-bar when the FROPS is fully raised (safe configuration);
(3) lowering the roll-bar when the FROPS is not fully raised nor fully lowered.

Consequently, to measure the torque values needed in these situations, the corresponding three lever arms were calculated considering the output of the previous analysis:

- l_1_ representing the distance between the hinge point and the point where the operator’s force is applied to raise the roll-bar in case of fully lowered FROPS.
- l_2_ representing the distance between the hinge point and the point where the operator’s force is applied to lower the roll-bar in case of fully raised FROPS.
- l_3_ representing the distance between the hinge point and the point where the operator’s force is applied to lower the roll-bar when the FROPS is not fully raised nor fully lowered.

Accordingly, the limits of the moments exerted by the operator on the FROPS in the worst situations are the following:

- M_MAX1_ = (F_OECD_ + 25%) × l_1_ (raising from the rest configuration)
- M_MAX2_ = (F_OECD_ + 25%) × l_2_ (lowering from the rest configuration)
- M_MAX3_ = (F_OECD_ + 50%) × l_3_ (lowering when the FROPS is not fully raised nor fully lowered).

**Figure 8:**
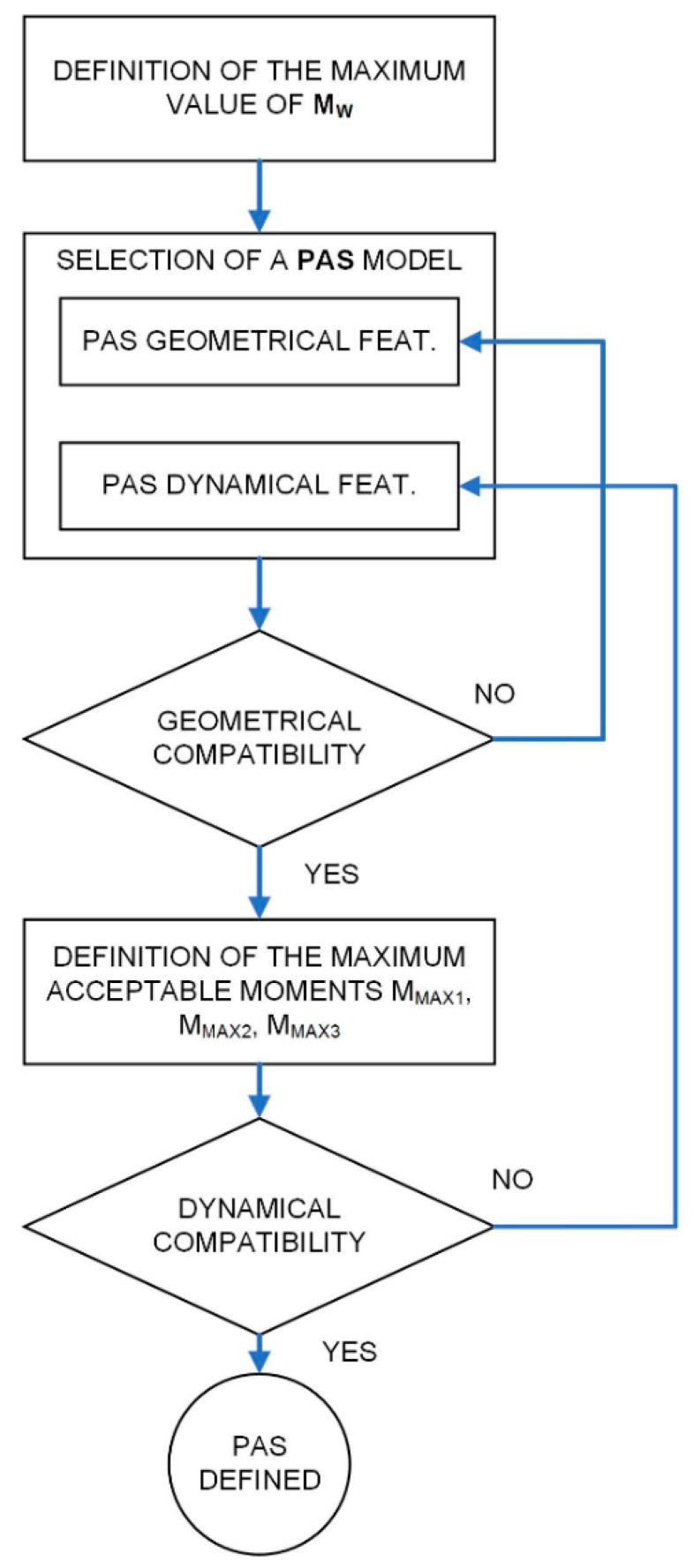
Scheme of the PAS selection procedure.

Based on the above considerations, a procedure (schematized in Figure 8) for the proper PAS dimensioning and selection can be derived, consisting of the following steps:

1. Definition of the maximum value of M_W_ (rest configuration of the FROPS).
2. Selection of a PAS model taking into account the following features:
  - PAS geometrical features;
  - PAS dynamical features (exercisable force in raising/lowering operations);
3. Verification of the geometric compatibility of the PAS;
4. Definition of the forces’ limits and the related acceptable moments in worst situations calculating MMAX1, MMAX2, and MMAX3 as suggested above.
5. Definition of the combined effects of the operator, the PAS, and the FROPS weight in the three worst situations suggested above:
  - M_OP1_ = M_W_ − M_PAS_, i.e., the moment exerted by the operator in raising the FROPS supported by the PAS when the FROPS is fully folded (rest configuration);
  - M_OP2_ = M_PAS_ − M_W_, i.e., the moment exerted by the operator in lowering the FROPS supported by the PAS when the FROPS is fully raised (safe configuration);
  - M_OP3_ = M_W_ − M_PAS_, i.e., the moment exerted by the operator in lowering the FROPS supported by the PAS when the FROPS is not fully raised nor folded.
6. Verification of the forces’ compatibility, comparing the combined effects of the operator, the PAS, and the FROPS weight in the three worst situations to M_MAX1_, M_MAX2_, and M_MAX3_ respectively, where the following conditions should be satisfied:
  - M_MAX1_ ≤ M_OP1_
  - M_MAX2_ ≤ M_OP2_
  - M_MAX3_ ≤ M_OP3_

#### 4.3.2. Geometrical Compatibility 

The further step of the PAS development consisted in dimensioning both the FROPS and the PAS and verifying their compatibility by means of CAD modeling. In particular, the FROPS’ roll-bar is made of steel tubulars with a squared hollow section (70 mm × 70 mm and thickness 5 mm). Its main features are: height from the hinge joint 1210 mm and weight 72.37 kg. Such a FROPS can equip a tracklaying tractor having an unballasted mass up to 3500 kg. Due to the forces involved in these operations, a gas-spring system was chosen. 

**Figure 9:**
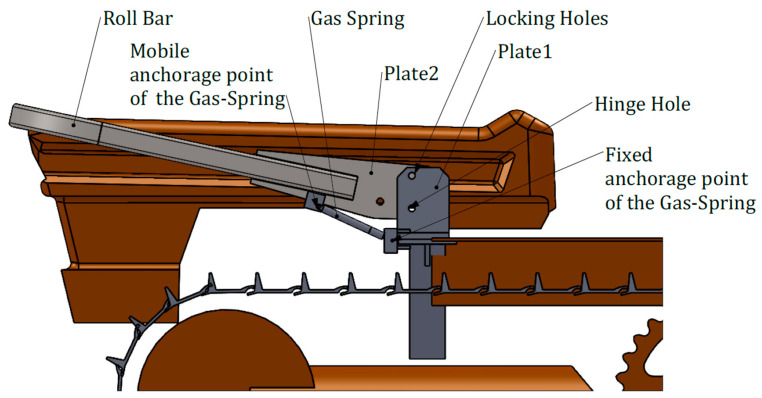
Features of the anchorage points of the PAS (lateral view).

Firstly, the characteristics of the anchorage points were determined by means of CAD tools (Figure 9).

**Figure 10:**
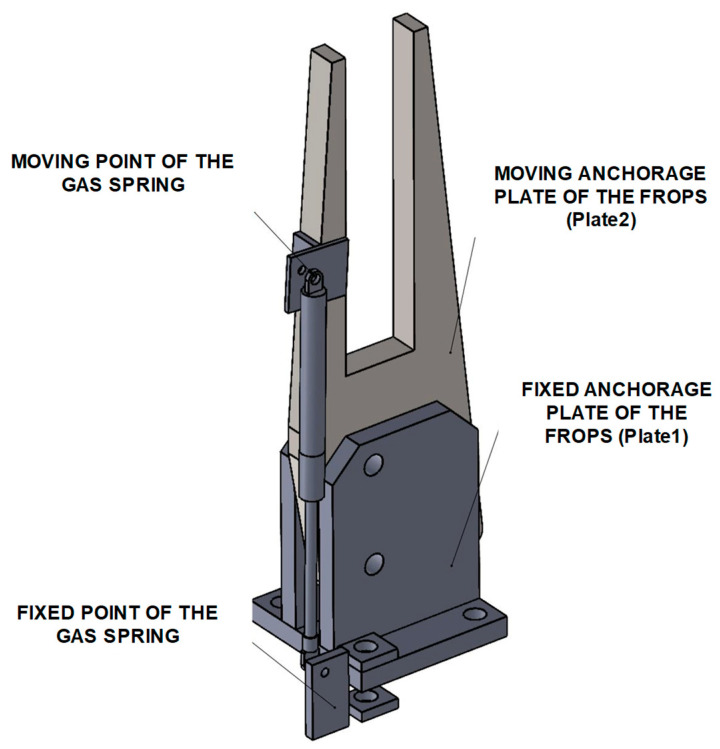
Details of the gas-spring assembling points.

In more detail, the hinge joint consists of two parallel plates (Plate1 in Figure 9) anchored to the mountings of the ROPS and a movable plate (Plate2 in Figure 9) inserted between them. The roll-bar is essentially made by square section tubes, welded together and with Plate2. Plate1 has two holes, the lower hole is for the hinge pin, the upper one is used to lock Plate2, and so the roll-bar in the rest or safe configuration by means of a movable pin. In Figure 10, the details of the gas-spring assembling points are shown.

Once the moving point of the gas spring, its extended length, and its stroke are defined, the fixed point can be determined. 

#### 4.3.3. Dynamical Compatibility

To select the gas spring model the dynamical analysis of the PAS was performed. Firstly, M_WMAX_ has to be considered, which corresponds to the maximum moment of the FROPS due to the weight force calculated using the following equations where P is the weight of the FROPS; d represents the distance between GC and the hinge axis; α is the folding angle that in this case is equal to 0° degrees (FROPS fully lowered):

Mw = P × b(3)

**Figure 11:**
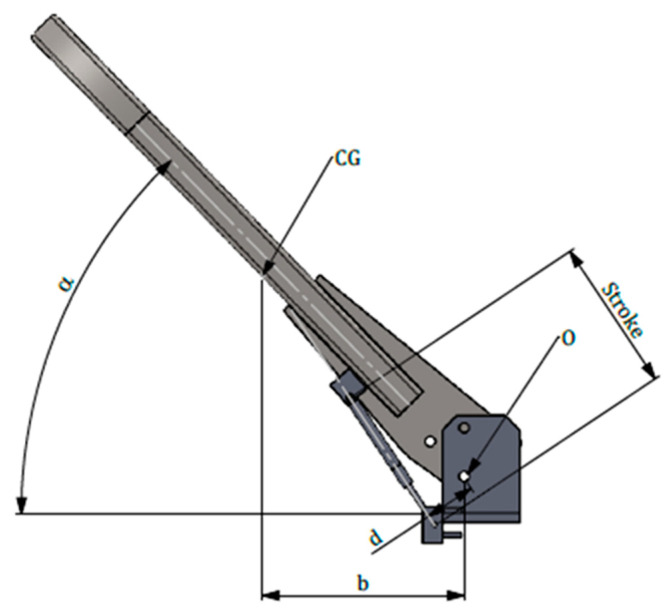
Main features of the PAS.

This value allows the preliminary definition of the gas-spring model since the gas-spring nominal force (F_PAS_) can be derived from the following equation:

F_PAS_ = (P × b)/(d) (4)

where (P × d) is the maximum value of Mw as defined above, while b is the lever arm of the spring (i.e., the distance between the hinge point of the FROPS and the anchoring point of the gas-spring on the FROPS) as shown in Figure 11. The value of F_PAS_ is the sum of the force exerted by the gas springs fitted on each side: if two gas springs of equal force are applied, the value of F_PAS_ shall be divided by two; in the case of two gas springs with the same geometrical features and different forces, their sum shall be at least equal to F_PAS_. In our case study, two equal gas springs were chosen, which satisfy the above criteria. In detail, a couple of gas springs having the following main characteristics was selected: minimum force: 1150 N; stroke: 146 mm; extended length: 385 mm; and a ratio of 1.32.

It has to be noted that since a couple of gas springs is used (one gas spring for each FROPS mounting), the moment exerted by the gas-springs (M_PAS_) is the sum of the moment of each gas spring:

M_PAS_ = (F_PAS_ × 2) × d (5)

Besides, the limits of the moments that can be exerted by the operator on the FROPS in the worst situations are the following:

- M_MAX1_ = (75 N + 18.75 N) × 1.150 m = 107.81 Nm (raising operation from the rest configuration).
- M_MAX2_ = (75 N + 18.75 N) × 0.596 m = 55.88 Nm (lowering operation from the safe configuration).
- M_MAX3_ = (75 N + 37.5 N) × 0.803 m = 90.34 Nm (lowering operation when the FROPS is not fully raised nor fully lowered: in this case, a folding angle of 45° should be considered).

To verify the dynamic compatibility of the PAS, these values were compared to the moments determined by the FROPS’ weight (M_W_) and the moments exerted by the selected gas-springs (M_PAS_) calculated for different FROPS’ positions (i.e., different values of the folding angle α), as shown in Table 3.

**Figure 12:**
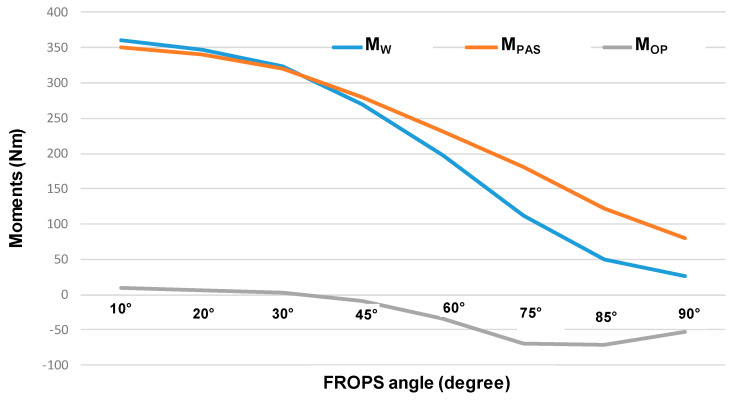
Variation of the moments involved in the FROPS handling in relation to the FROPS angle.

Additionally, in the last two columns of Table 3, the values related to the balance of the moments (M_OP_ = M_W_ − M_PAS_) and the limits of the moments (M_MAX_) are reported, where the former represents the practical loads the operator has to deal with to operate the FROPS for a certain angle (M_OP_), while the latter is the maximum theoretical load the operator can bear (M_MAX_) avoiding excessive stress and discomfort. The comparison between them has to be made considering the absolute values of moments, while the positive values of the moments represent the ones concordant to the lifting angle. The torques behavior is schematized in Figure 12, where M_W_, M_PAS_, and M_OP_ are compared for different FROPS angles.

### 4.4. Validation

**Figure 13:**
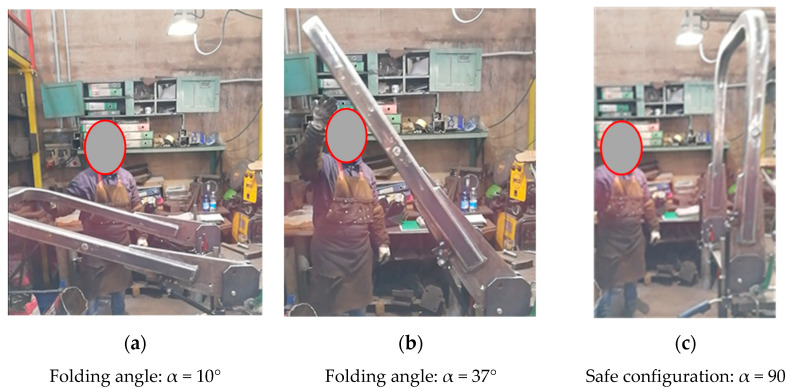
Practical tests concerning the raising operations.

A prototype was manufactured and practical tests were carried out in the laboratory (Figure 13). From this analysis, it was confirmed that after 27° of rotation (corresponding to a folding angle α = 37°) the PAS is able to complete the raising phase by itself (Figure 13b), i.e., no further efforts by the operator are needed. In addition, the gas springs are able to keep the FROPS in the safe configuration (Figure 13c) and the operator can lock the FROPS by means of the lateral joints safely (e.g., it might happen that if the gas springs are too weak, the FROPS might fold down due to an improper maneuver of the operator, who can be hit by the mounting).

**Figure 14:**
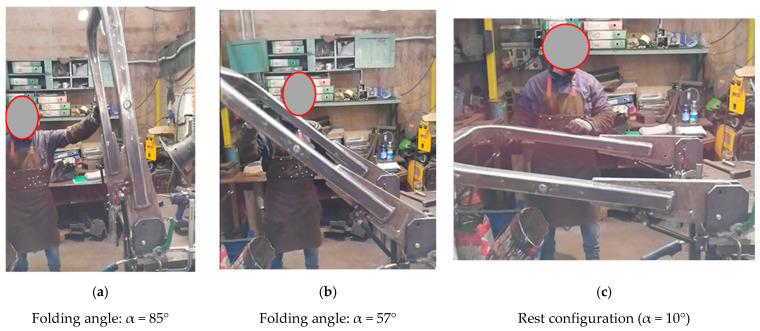
Practical tests concerning the lowering operations.

Conversely, in the initial stage of the lowering phase, the value of the force needed to lower the FROPS is slightly higher than the maximum value foreseen by the OECD Code no. 6 (Figure 14a), but the corresponding moment is below the limit value calculated above. After that, the lowering phase is not strenuous for the operator since the operating force to handle the roll-bar decreases up to the zero value near the rest configuration thanks to the weight force (Figure 14b,c).

In addition, it is noteworthy mentioning that the combined effects of M_W_ and M_PAS_ assure a reduced rotation speed of the FROPS, diminishing the risk that the operator can be hit during lowering/raising operations. The inclusion of additional plates to fix the PAS avoids the reduction of the structural strength of the FROPS, enabling the installation of such a system also on already-in-use tractors. It should also be remarked that the installation of this type of PAS on already-in-use FROPS has a cost of about EUR 150, while its implementation by the tractor manufacturer—new novel models—can be much cheaper. Moreover, this cost is at least ten times cheaper than the cost of an automated system.

**Figure 15:**
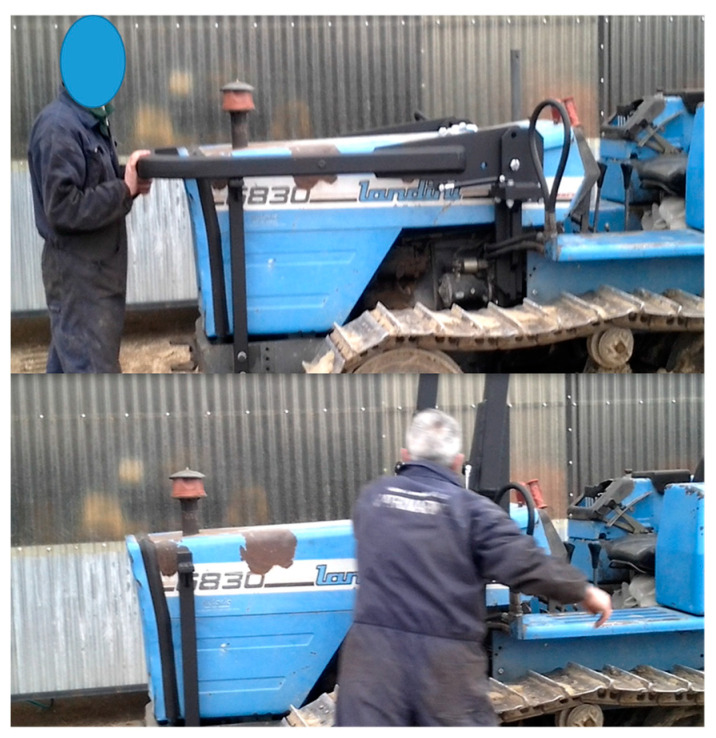
Practical tests of usability by farmers.

Finally, it has to be noted that the PAS was installed on a tracklaying tractor to test its practical usability. For this purpose, two tractor users were asked to use the tractor and operate the FROPS several times to mimic real infield operations (Figure 15). The first feedback from the operators was positive since they declared that the PAS makes the FROPS handling easier for both operations (raising and lowering) and they confirmed that if this system will be available on the market in the future, they will be eager to apply it to their tractors. Therefore, although the number of interviewed farmers is not large, these preliminary tests can be considered valid as they are in line with similar tests among farmers on the FROPS’ use, such as the research by Cremasco et al. [39]. 

## 5. Discussion

The achievement of safe human interaction with technical systems requires the integration of safety and ergonomic issues in the design and management of work equipment in a practical manner [40,41]. Following such a research cue, the current study was aimed at investigating the problem of the use of tractors equipped with front-mounted foldable protective structures. In particular, it was found that, although safety regulations concerning work equipment require that they should be designed taking into account the risks of accidents arising from their foreseeable misuse, a few studies have investigated the FROPS’ misuse providing technical solutions to reduce this occurrence. Accordingly, the implementation of a PAS was carried out taking into account the users’ behavior. As a result, this analysis allowed us to achieve the following outputs.

A procedure to support the definition of the grasping points of front-mounted FROPS for tracklaying tractors was developed, reducing the lack of technical references on the ergonomic handling of foldable structures for this type of tractors. The definition of these points was obtained by merging practical experience and CAD modeling considering the worst situations when handling the FROPS. Such an approach is in line with other studies [42,43] fostering the inclusion of worst-case scenarios when designing products focusing on the human factors perspective. Starting from this point, the acceptable limits of the moments that the operator can exert on the FROPS were determined, providing a reference basis for the definition of the acceptable limits of forces and moments involved in handling the FROPS. In such a context, it is noteworthy to mention that focusing only on the handling force limits provided by the OECD Codes when selecting the PAS dynamic features might lead to an unsafe situation when the fully raised FROPS has to be blocked. Therefore, a more thorough analysis that includes the evaluation of the moments exchanged between the operator on the one hand, and the system (i.e., the combination of the FROPS and the PAS) on the other, during all handling operations, is necessary. Additionally, another finding of this study consists in the definition of a grasping area adapted to tracklaying tractors since the operator’s behavior when handling the FROPS differs from that in the case of wheeled tractors. 

Based on these considerations, we believe that such outputs can contribute to expanding knowledge on ergonomic features of protective structures destined to equip tractors to be used not only in the agricultural context but also in other sectors where this type of machinery is common (e.g., construction and mining works) [44]. Such a finding can also contribute to involve human factors in safety research on agricultural machinery by means of procedures aimed at reducing workers’ OHS risks [45].

Moreover, this study allowed us to implement a procedure for the selection of a PAS for the FROPS that can also be used at a practical level to update already-in-use tractors, providing a feasible solution both from the technical and economical point of view, responding to the research needs outlined by several studies [46,47]. The results achieved, accomplish the findings of Caffaro et al. [48], who pointed out the need to consider both the user and producer standpoint to augment the safety level of dangerous work equipment. In addition, it has to be stressed that researching on ergonomic and safety issues of farmers represents a step forward to augment the social features of sustainable agricultural systems, in line with recent research trends [49,50]. 

Finally, it is worth noting that to augment the validity of the proposed procedure, additional applications on different tractor types are needed. In particular, this can improve the reliability of the criteria proposed for the definition of the grasping points of the FROPS mounted on tracklaying machinery, on the one hand, refining the selection process of the gas-spring models to further reduce the operator’s efforts in the lowering phase without compromising the safety level on the other. In addition, practical tests with a larger group of operators are also needed in order to better analyze the usability and practicality of the system.

## 6. Conclusions

The study analyzed the use of foldable protective structures for tractors with the goal of providing a partial assistance system aimed at reducing their misuse by the operators. Accordingly, a procedure for the proper PAS selection was proposed and verified by means of a practical case study on a tracklaying tractor. The results achieved can contribute to expanding knowledge on technical solutions aimed at improving the human-machinery interaction in the agricultural sector. However, although such an output is more relevant in this sector, it can also contribute to advance scientific knowledge on the improvement of machinery safety and ergonomics in other domains. Therefore, further research is expected to extend the validity of the research findings beyond the analyzed case study.

## Figures and Tables

**Table 1:** Acceptable force limits foreseen by the OECD Code no. 6 for raising/lowering operations.

Zone	I	II	III
Acceptable force (N)	100	75	50

**Table 2:** Examples of FROPS weights extracted from [11].

OH [mm]	H_GC_ Height [mm]	P [kg]
1100	487	70.5
1200	515	72.4
1300	544	74.3
1400	575	76.4

**Table 3:** Moments’ comparison table, where the last column indicates the limits that should not be exceeded.

α [deg]	b [m]	M_W_ [Nm]	d [m]	F_PAS_ [N]	M_PAS_ [Nm]	Stroke [mm]	M_OP_ [Nm]	M_MAX_ [Nm]
10	0.466	360.14	0.114	1531.15	350.39	249.53	9.75	107.81
20	0.449	346.99	0.116	1470.45	340.32	269.67	6.66	90.34
30	0.419	323.29	0.114	1410.01	320.36	289.73	2.93	90.34
45	0.349	269.64	0.105	1323.23	279.16	318.52	−9.52	90.34
60	0.256	197.62	0.093	1244.72	231.25	344.57	−33.64	90.34
75	0.145	112.12	0.077	1177.51	181.35	366.87	−69.23	90.34
85	0.065	50.5	0.054	1133.4	125.74	379.28	−75.24	90.34
90	0.035	27.02	0.035	1150	80.50	384.18	−53.48	55.88
